# Research progress on the roles of extracellular vesicles in tumor immunity and drug resistance

**DOI:** 10.3389/fimmu.2026.1750226

**Published:** 2026-03-11

**Authors:** Minghong Zhao, Meijin Liu, Wenlong Huang, Haibin Shen, Qing Jin, Tao Qin, Defa Huang

**Affiliations:** 1 Laboratory Medicine, Guizhou Aerospace Hospital, Zunyi, China; 2 Laboratory Medicine, People’s Hospital of Ganzhou Economic Development Zone, Ganzhou, China; 3 Department of General Medicine, The First People’s Hospital of Zunyi (Third Affiliated Hospital of Zunyi Medical University), Zunyi, China; 4 Laboratory Medicine, The First Affiliated Hospital of Gannan Medical University, Ganzhou, China

**Keywords:** biomarkers, drug resistance, extracellular vesicles, tumor immunity, tumor microenvironment

## Abstract

Extracellular vesicles (EVs) have emerged as pivotal mediators of intercellular communication and have attracted considerable scientific interest in recent years owing to their critical roles in tumor immunity and drug resistance. This review offers a comprehensive overview of the mechanisms by which EVs function within the tumor microenvironment, focusing on their involvement in immune evasion, tumor progression, and the development of resistance to therapeutic agents. By summarizing recent research advances, this review highlights the potential of EVs as diagnostic biomarkers and therapeutic targets and emphasizes their significance in improving treatment efficacy and overcoming clinical resistance. This review also outlines future research directions to clarify the multifaceted roles of EVs in cancer biology and facilitate the development of novel therapeutic approaches to enhance patient outcomes.

## 1 Introduction

Cancer poses a major threat to human health and life span. Biological processes in tumors and metabolism significantly affect the immune system. Cancer cells avoid detection by the immune system and even use it for their own growth under specific conditions (1–3). Therefore, a comprehensive understanding of the complex interactions between tumor cells and the microenvironment is necessary to identify the causes of tumor growth. Extracellular vesicles (EVs), are nanometric particles produced by various living cells and are characterized by a bilayer membrane structure (4). Currently, no single marker can distinguish between different types of EVs. Therefore, the International Society for Extracellular Vesicles categorizes EVs based on their size: Small EVs <200 nm in diameter and large EVs (including microvesicles [MVs] and apoptotic bodies) >200 nm in diameter (5, 6). They are present in different body fluids, such as blood, urine, ascites, saliva, cerebrospinal fluid, and bronchoalveolar lavage fluid (7). They are involved in key cancer biology areas such as tumor immunity and drug resistance. Tumor-derived EVs (TEVs) carrying immune-active substances facilitate communication among immune cells around the tumor, thereby altering the TME. For instance, TEVs carrying immunosuppressive factors inhibit T-cell activation, thereby promoting immune evasion (8). Within the tumor microenvironment, virtually all cellular constituents, including tumor cells, immune cells (e.g., T cells, B cells, natural killer cells, dendritic cells, and macrophages), and stromal cells (e.g., cancer-associated fibroblasts and endothelial cells), are capable of secreting EVs that carry distinct molecular cargo (9, 10). EVs derived from diverse cellular sources collectively constitute a sophisticated “vesicle-mediated communication network” (11, 12). By conveying highly specific molecular signals, these EVs exert profound and heterogeneous regulatory effects on multiple facets of immune cell biology, including recruitment, activation, differentiation, functional modulation, and exhaustion, thereby playing a dual role in tumor immunity (13). Specifically, they can either suppress antitumor immunity by delivering inhibitory signals that foster immune tolerance and tumor progression or enhance antitumor immunity by transmitting stimulatory signals that potentiate immune cell activation and effector responses (14). This functional duality critically influences tumor immune surveillance, immune evasion, and immunotherapy outcomes. Therefore, understanding the dynamic roles of EVs in tumor immunity and drug resistance is important for developing novel therapies.

This review summarizes the mechanisms of action of EVs in the TME, highlighting their roles in tumor immune evasion, progression, and treatment resistance. This review also outlines the future directions and challenges of EV research in oncology.

## 2 EVs of different cellular sources regulate tumor immunity

**Figure 1:**
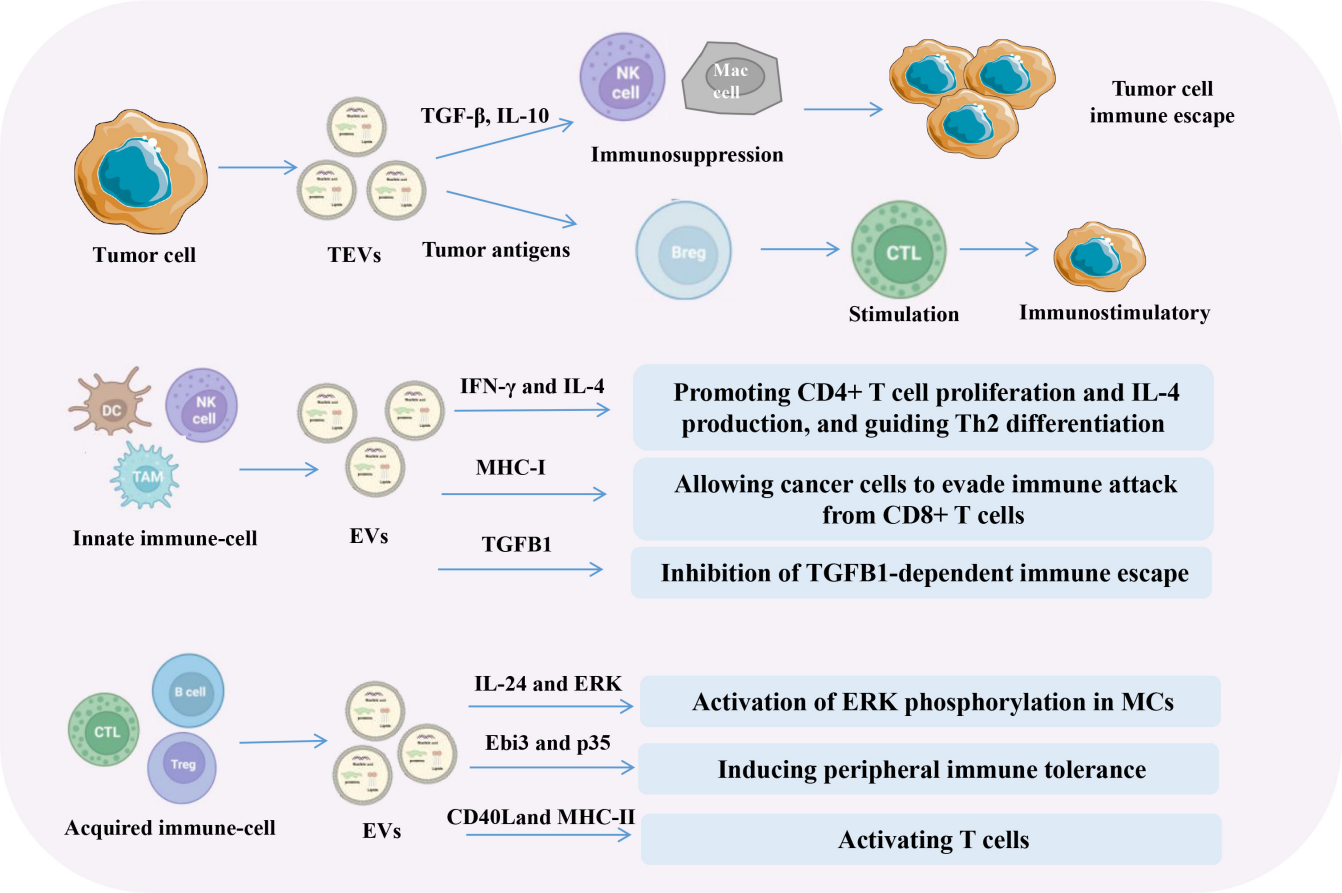
The roles of EVs from different cell sources in tumor immunity. Tumor cells, innate immune cells, and acquired immune cell-derived EVs exert inhibitory or activating effects on the immune system through various bioactive substances. Tumor cells, immune cells, and other related cells play dual roles in both enhancing tumor immunity and helping the tumor evade the immune response.

EVs contain immunomodulatory bioactive substances that play an essential role in altering the complex network of immune tumors. EVs carrying bioactive molecules mediate intercellular communication in the tumor microenvironment (TME) and regulate key processes such as immune responses, angiogenesis, and pre-metastatic microenvironment formation (15). However, because different EVs exhibit different functional features, their involvement in cancer is complex and context dependent. This diversity of EVs and their functions contribute to the complexity and ambiguity of their anti-tumor and pro-tumor effects (**Figure 1**). Research on the biological functions of EVs has shown that they play different roles in various cell types, impacting cancer development and treatment effectiveness. They can either promote or inhibit tumor growth. The immune-related effects of tumor-associated EVs are complex and variable. They primarily function via intercellular communication pathways.

### 2.1 TEVs affect tumor immunity

TEVs play crucial roles in the tumor immune response process, mainly by regulating the immunomonitoring of tumor cells through three methods. TEV presents different antigens and immune inhibitory molecules, influences the interactions between tumor immune cells, and changes the TME. Tumor cells avoid immune detection by hiding, forming suppressor cells, controlling antigen presentation, and impacting the proper functioning of the immune system.

Recent literature suggests that TEVs have potent immunosuppressive effects. Upon prolonged exposure to TEVs, immune cells may favor tumor growth, creating an environment conducive to tumor growth and affecting the anti-cancer responses in the body. Cancer-derived EVs inhibit CD8+ T cell proliferation and activation, promote regulatory T cell and myeloid-derived suppressor cell expansion, suppress NK cell activity, interfere with monocyte differentiation (16), and express molecules such as PD-L1 and TGF-β that mediate immunosuppression (17, 18). Wu et al. showed that EVs obtained from patients with clear cell renal cell carcinoma prevent the activity of tumor-infiltrating NK cells by upregulating TGF-β/SMAD signaling, implying that TEVs regulate the activity of immune cells in the TME (19). These actions create a conducive environment for tumor growth and contribute to drug resistance in cancer therapy (20). Although EVs are often linked to tumor immune evasion and resistance, they also exert antitumor effects by displaying tumor antigens, activating T cells, or transporting the stress protein heat shock protein (HSP)-70 to stimulate NK cells. Moreover, TEVs have demonstrated potential as cancer vaccines in animal studies, triggering antigen-specific responses from T and B cells. This indicates that immune responses to TEVs may vary depending on the context, affecting whether they enhance or inhibit such immune responses (21).

From the standpoint of innate immunity, EVs released by tumor cells may carry immunosuppressive molecules, such as TGF-β and interleukin (IL)-10, which impair the activity of innate immune cells, such as macrophages and NK cells. Plebanek et al. discovered that EVs produced by melanoma cells cause an innate immune reaction by drawing NK cells and triggering TNF-related apoptosis-inducing ligand-induced cell lysis to initiate immune vigilance and strengthen the inherent antitumor immunity. This leads to the destruction of cancer cells before they can spread and markedly reduces the incidence of lung metastasis (22). This suppression helps tumor cells evade immune system attacks. Moreover, these EVs may contain molecules that modulate inflammation, thereby affecting inflammatory conditions within the tumor and influencing the behavior of immune cells. The TME is initially characterized by an immune-activating setting with increased proinflammatory signals during early tumor development, in which immune cells generally exhibit a pro-inflammatory profile. However, as tumor growth and metastasis progress, the TME gradually shifts to a hypoxic state featuring low pH, decreased glucose levels, higher fatty acid concentrations, reduced amino acid availability, elevated adenosine levels, and increased lactate levels. This persistent inflammatory condition contributes to its initiation and progression (23). Importantly, EVs secreted by tumor cells are crucial for maintaining this microenvironment. In patients with brain metastases, elevated levels of cell migration-inducing and hyaluronan-binding proteins have been detected in both tumor tissues and EVs, which further promote endothelial cell branching and inflammation in the perivascular microenvironment (24). In addition, EVs can carry chemokines and cytokines that attract immune cells to the tumor site. Although this recruitment can sometimes strengthen immune responses against tumors, it can also be exploited to divert immune cells by conveying information about immune evasion tactics, such as avoiding immune antigens or other molecular mechanisms of immune escape. This allows tumor cells to effectively evade detection by the innate immune system. For example, cancer cells inhibit NK cell proliferation and cytotoxicity by downregulating NKG2D expression. TEVs can also induce increased levels of nitric oxide synthase-2, suppress mitochondrial oxidative phosphorylation, and enhance glycolysis through nuclear factor (NF)-κB-dependent metabolic alterations. These changes result in higher PD-L1 expression in macrophages, elevated glucose transporter-1 levels in primary tumors, increased expression of YKT6 associated with vesicle secretion, and greater infiltration of immunosuppressive macrophages into the pre-metastatic niche (25). Khenchouche et al. found that EVs expelled from gastric cancer cells trigger the NF-κB signaling pathway in macrophages, resulting in the discharge of pro-inflammatory cytokines that sustain the inflammatory TME, thereby allowing gastric cancer progression (26). CircRNA circCCAR1 is transported by EV originating from HEK cells and stabilizes PD-L1 in CD8+ T Cells by interacting with the miR-127-5p/WT1-associated protein regulatory loop, thereby supporting hepatocellular carcinoma growth, metastasis, and immunosuppression (27).

In terms of adaptive immunity, TEVs carry tumor-specific antigens that are recognized by the immune system, thereby initiating an adaptive immune response. TEVs are essential for helping T and other immune cells identify and destroy tumor cells. They deliver tumor antigens to antigen-presenting cells (APCs), activating T cell responses and potentially boosting immune memory, which enhances the ability of the immune system to react to tumor antigens (28). However, TEVs can dampen the immune response by transferring immune checkpoint molecules to T cells, leading to T cell exhaustion. In the context of immunotherapy, TEVs containing immune checkpoint-associated molecules can impact the efficacy of immune checkpoint inhibitors (28). Furthermore, TEVs induce T-cell apoptosis via Fas ligand (FasL), inhibit T-cell receptor (TCR) function, and modify the gene expression patterns of Tregs and effector T cells, with TGFB1 encouraging the development of Tregs (29).

### 2.2 Innate immune cell-derived EVs affect tumor immunity

Immune cells secrete chemokines via EVs, attracting immune cells to the TME. This is important for coordinating local immune responses, determining the location of all immune cells in three-dimensional space, and initiating intercellular communication within the TME. EVs from immune cells exert immunomodulatory effects similar to those of their parent cells; however, their functions depend on the cell type. In particular, EVs from innate immune cells significantly affect tumor immunity by facilitating interactions with immune elements. EVs generated by innate immune cells may exert diverse effects on antitumor immune responses, facilitating immune evasion and promoting immune activation and effector functions. The next section discusses these dual roles in detail, highlighting the roles of different types of innate immune cells (**Table 1**).

**Table 1:** Immunomodulatory effect of EVs derived from innate immune cells.

EVs source	Mediator	Target cells	Related key molecules	Biofunctions	References
DCs	OX40L	T cells	IFN-γ and IL-4	Promoting CD4+ T cell proliferation and IL-4 production, and guiding Th2 differentiation	(30)
	–	SK-BR-3 breast cancer cell	IFN-γ	Increasing IFN-γ production from CD3+ T cells	(31)
	HSP70 and HSP90	T cells	MHC-I、MHC-II and CD86	Activating CD4+ and CD8+ T cells	(32)
	MHC-II, ICAM-1	T cells	–	Inducing a strong adaptive immune response	(34)
	TNF-α	B cells	NF-κB	Inducing inflammatory response	(37)
TAMs	miR-21-5p and miR-29-3p	T cells	STAT3	Inducing Treg/Th17 imbalance and promoting the progression and metastasis of epithelial ovarian cancer cells	(40)
	PD-L1	T cells	–	Inhibiting the proliferation and activity of CD8+ T cells	(41)
	LINC01592	cancer cells	E2F6/NBR1/MHC-I	Allowing cancer cells to evade immune attack from CD8+ T cells	(42)
	miR-21	T cells	PEG3	Accelerating immune escape from glioma	(43)
NK cells	miR-186	Neuroblastoma cell line	TGFB1	Inhibition of TGFB1-dependent immune escape	(45)
MCs	CD63 and OX40L	T cells	OX40	Promoting Th2 cell proliferation and differentiation	(47)
Neutrophils	miR-30d-5p	macrophage	NF-κB	Increasing polarization ratio of M1 macrophages and pyroptosis of macrophages	(50)
MDSCs	miR-29a-3p and miR-93-5p	T cells	T-bet and STAT3	Inhibit Th1 and Th17 cell differentiation	(53)

Dendritic cells (DCs), a type of APCs, absorb antigens and convert them into peptides. These peptides are presented to T cells via major histocompatibility complex (MHC) molecules on the cell surface, leading to an adaptive response. DCs produce EVs in response to thymic stromal lymphopoietin, which contains OX40L. OX40L supports the proliferation of CD4+ T cells, increases IL-4 production, and induces the differentiation of Th2 cells (30). DC-derived EVs also enhance the production of interferon-γ by CD3+ T cells upon interaction with SK-BR-3 breast cancer cells (31). Moreover, DCs release different types of EVs with various components, such as HSP70 and HSP90. They activate CD4+ and CD8+ T cells, MHC-I, MHC-II, and CD86 (32). DC-derived EVs further stimulate the innate immune system by activating NK cells via ligands for NKG2D-L and IL-15Rα (33). In addition to stimulating T cell immune responses and DC-derived EVs, T cells are activated indirectly through the presentation of antigen–MHC complexes and by delivering intercellular adhesion molecule-1 molecules to subpar APCs, such as B cells (34). Activated class B cells produce and secrete EVs that effectively stimulate CD4 and CD8 T cells, inducing highly potent antitumor immune reactions (35). DC-derived EVs induce immune responses via the EV-induced release of inflammatory markers. When DCs express alpha-fetoprotein on EVs, this initiates a chain reaction leading to CD8+ T cell activation through a considerable increase in interferon-γ and IL-2 production, followed by a reduction in inhibitory factor levels, such as IL-10 and TGF-β levels, in T-cells, such as CD25+ Foxp3+ Tregs (36). EVs transporting TNF-α produced by DCs show robust interactions with NF-κB in B cells, thereby boosting inflammatory responses in human umbilical vein endothelial cells (37).

Macrophages are immune cells that develop in the bone marrow. They participate in both natural and adaptive immune responses in the body. Macrophages and EVs perform different functions owing to their different sources. For example, EVs derived from lipopolysaccharide-activated RAW264.7 macrophages provide microglial protection (38). EVs derived from Schwann cells containing MFGE8 (39) provide neuroprotective benefits by promoting microglial polarization toward the immunosuppressive M2 phenotype. Tumor-associated macrophages (TAMs), primarily M2 type macrophages and are important components of the TME. They exert no cytotoxic effects, promote tumor cell growth through growth factors, and exhibit immunosuppressive activity. Recent studies have revealed the role of TAM-released EVs in helping cancer cells evade host defenses. EVs produced by M2 macrophages contain miR-155-5p, which interferes with the mechanism by which ZC3H12B ensures that IL-6 levels are steady, thereby facilitating immune evasion and growth of colon cancer cells. Furthermore, TAM EVs express miR-21-5p andmiR-29-3p, which disrupt the Treg/Th17axis via signal transducer and activator of transcription 3 signaling and drive the progression and metastasis of epithelial ovarian cancer (40). TAM EVs are also packed with PD-L1, which blocks the development and function of CD8+ T-cells (41). Studies on the tissues of patients with melanoma have shown that TAM EVs, especially those from CD163+ TAMs, hinder the functioning of CD8+ T cells. Furthermore, TAM-secreted EVs containing long non-coding RNAs LINC01592 upregulate *NBR1* gene transcription in tumor cells by directly binding to E2F6. It then acts on MHC-I proteins. After binding to NBR1 proteins, ubiquitin and MHC-I proteins are degraded and form autophagic lysosomes, which reduce MHC-I surface expression on tumor cells. Targeting the E2F6/NBR1/MHC-I pathway with small interfering RNA or specific antibodies significantly reduces the tumor-promoting effects of LINC01592 and M2-TAM-driven tumor growth (42). EVs originating from M2 macrophages in bone tissue can prevent the growth and cell-killing activity of CD8+ cells by controlling miR-21/PEG3 expression, thereby allowing immune evasion in glioma (43).

NK cells were identified in 1976 as part of the first line of defense of the immune system. Innate immune cells protect against harmful microorganisms. Research on patients with cancer has shown that EVs obtained from NK cells carry distinct NK cell markers, such as CD56, NKG2D, CD94, and CD140, along with cytolytic effector molecules, such as FasL, perforin, and granzyme (44). They directly affect cancer cells, stopping their escape; cancerous tissues mainly interact with them, demonstrating the cytotoxic effects of NK cells (14). They also allow EVs produced by NK cells that have already encountered and responded to neuroblastoma to teach other unexposed NK cells to ensure a better and stronger action against neuroblastoma cells. Neuroblastoma NK cell-derived EVs with tumor suppressor miR-186 stop TGFB1 from helping the tumor to hide and decrease tumor growth and spread (45).

Mast cells (MCs) are the main effectors of both early and late allergic inflammation mediated by IgE. These cells are activated once FcϵRI receptors attach to IgE antibodies linked to multivalent antigens. They release both stored and freshly prepared lipid mediators and cytokines. The various biological effects of mediators play important roles in the functions of MCs in acute allergic responses and chronic allergic inflammation. MCs are also involved in various other inflammatory processes and serve as triggers for IgE-related events. For example, MCs or MC secretions play a role in the recruitment of leukocytes to the site of inflammation, antigen presentation by T cells, interactions with cells of the adaptive immune system, and tissue remodeling (46). Studies have shown that mast stem cells originating from the bone marrow secrete EVs that express CD63 and OX40L. Moreover, OX40L expressed by these EV binds to OX40R on T cells, promoting the growth and differentiation of Th2 cells (47). Additionally, EVs from the MC line HMC-1 were taken up by the lung cancer cell line A549. Uptake of the KIT protein activates the KIT–stem cell factor signaling pathway, increasing the expression of cyclin D1 and promoting the proliferation of human lung adenocarcinoma cells (48). Neutrophils are an important part of the immune system, protecting the body from getting sick by eating (engulfing) and eliminating germs; they are also needed for the proper function of other parts of the body (49). For example, miR-30d-5p in polymorphonuclear neutrophil-derived extracellular vesicles promotes M1 macrophage polarization and initiates macrophage pyroptosis via NF-κB activation (50).

Myeloid-derived suppressor cells (MDSCs) are specialized pathological myeloid cells that exert potent immunosuppressive effects and are generated under various disease conditions. These cells are of two types: granulocytic or polymorphonuclear and monocytic MDSCs (51). EVs derived from MDSCs exhibit the immunosuppressive properties of their parent cells. They participate in tumor formation, new blood vessel generation, migration, and metastasis. Previous studies have shown that EVs produced by MDSCs contain miRNAs that affect MDSC function by altering myeloid cell differentiation and proliferation, promoting CD8+ T cell senescence, and blocking their proliferation (52). Additionally, these EVs impede the differentiation of Th1 and Th17 cells by targeting T-bet and signal transducer and activator of transcription 3 via miR-29a-3 p and miR-93-5p, respectively (53). Similarly, treatment with MDSC-derived EVs markedly decreased the proportion of M1macrophages (54).

### 2.3 Adaptive immunity cell-derived EVs affect tumor immunity

The regulation of adaptive immunity by innate immune cells is well established. Huang et al. reported that B cells producing IL-10 induced M2 polarization of macrophages in a melanoma tumor model (55). EVs from activated CD3+ T cells also activate resting CD3+ T cells by stimulating their proliferation (56). These results indicate that EVs from adaptive immune cells influence antitumor immune responses in many ways. This review discusses the EVs released from different subsets of adaptive immune cells and their effects on tumor immune responses (57) (**Table 2**).

**Table 2:** Immunomodulatory effect of EVs derived from acquired immune cells.

EV source	Mediator	Target cells	Related key molecules	Biofunctions	References
CLT	RAS/MAPK	MCs	IL-24 and ERK	Activation of ERK phosphorylation in MCs	(60)
Th cells	LAMP-1, TCR, and LFA-1; CD4, TCR, LFA-1, CD25, and Fas	CTL	–	Activating CD8+ T cells	(61)
Tregs	Let-7b, Let-7d, miR-155, miR-142-3p and miR-150-5p	Th cells	–	Inhibiting Th1 immune response and strengthen the inhibitory function of the immune system	(62)
	IL-35	T cells and B cells	Ebi3 and p35	Inducing peripheral immune tolerance	(66)
	CD39 and CD73	Th cells	adenosine	Inhibiting the activation and proliferation of CD4+ T cells	(67–69)
	miR-150-5p and miR-142-3p	DC	–	Decreasing secretion of IL-10 and IL-6	(70)
B cells	pMHC-II	T cells	–	Activating T cells	(72)

Adaptive immunity: T cells originate from the bone marrow, mature within the thymus, and enter the peripheral blood. These cells are essential for targeting antitumor immune responses. Helper T-cells are typically distinct from cytotoxic T-cells. These two types have different immune functions and surface markers. Helper T cells are CD4+ cells, whereas cytotoxic T lymphocytes are CD8+, CD4+, and CD8+ T cells. EVs induce DC apoptosis and T cell inhibition via different mechanisms involving peptide/MHC/TCR and intercellular adhesion molecule-1/lymphocyte function-associated antigen-1 (58, 59). Additionally, MCs internalized T cell-derived EVs within 24 h, resulting in the upregulated secretion of IL-24. Proteomic analysis has indicated the presence of a few types of proteins in these EVs, most of which are RAS/mitogen-activated protein kinase signaling-related proteins; this pathway induces extracellular signal-regulated kinase phosphorylation in MCs (60). CD4 becomes a co-receptor for TCR on helper T cells, facilitating interaction with MHC-II of APCs and promoting T-helper cell activation and differentiation. EVs from CD4+ helper T cells are characterized by typical EV markers, such as lysosomal-associated membrane protein-1, TCR, and lymphocyte function-associated antigen-1, and CD4-specific markers, such as CD4, CD25, and FasL. They enhance antitumor immunity by activating cytotoxic T lymphocytes (61).

Most Tregs are a group of CD4 T cells with immunosuppressive functions, recognized by the cell surface marker CD25 and high expression levels of the transcription factor Foxp3 (62, 63). Tregs modulate the immune system and promote self-antigen tolerance. Recent studies have shown that Tregs secrete EVs containing more membrane molecules than other T cell subsets (64). Treg-derived EVs deliver immunosuppressive molecules to target cells, similar to Tregs, with similar immunosuppressive effects on target cells (58, 65). IL-35 plays an important role in blocking T-cell activation, and the population of IL-35-rich Tregs is increased in the TME. IL-35 is released by Tregs upon activation with TCR to restrict infiltration and induce T cell death in the TME. EV IL-35 targets T/B cells and confers peripheral tolerance (66). EVs generated by Tregs expressing CD39 and CD73 inhibit CD4+ T cell activation and proliferation by promoting adenosine production (67–69). EVs released by Tregs contain various miRNAs, including Let-7b, Let-7d, miR-155, miR-142-3p, and miR-150-5p. These miRNAs play crucial roles in inhibiting Th1-type immune responses by suppressing the translation of target mRNAs, thereby enhancing immune suppressive functions of the immune system. Tregs pass let-7d to Th1 cells through EVs, inhibiting Th1 cell proliferation and cytokine production, thereby preventing systemic diseases (64). Moreover, the uptake of EVs enriched in miRNAs, such as miR-150-5p and miR-142-3p, by lipopolysaccharide-stimulated DCs decreases the secretion of IL-10 and IL-693 (70).

Unlike T cells, bone marrow B cells play important roles in host immune responses by secreting immunoglobulins, presenting antigens, providing costimulatory signals, and secreting cytokines to induce and modulate antitumor immunity (71). B and T cells interact with each other specifically via antigens during immune reactions. This is a conversation between pMHC-II on the surface of B cells and TCR on antigen-sufficient T cells. B cells can activate unique T cells in humans and mice because they contain pMHC and helper molecules. B cells release approximately 12% of pMHC-II daily in EVs, which activate T cells by binding pMHC-II to the TCR of CD4+ T cells (72). Generally, EVs from immune cells are important for communication between innate and adaptive immune cells and act as an intermediate link between the immune response and tumor cells.

### 2.4 EVs from other cellular sources act on tumor immunity

Fibroblasts, which are derived from mesenchymal cells, produce components of the extracellular matrix to form the tumor stroma. Fibroblasts located in the stroma of cancers, also known as CAFs, participate in the creation of the TME. Tumors can convert adjacent fibroblasts into CAFs. Activated CAFs promote tumor development, angiogenesis, invasion, metastasis, and extracellular matrix (ECM) remodeling outside the tumor site. Simultaneously, they are associated with resistance to chemotherapeutic drugs in tumors, thereby enhancing tumor proliferation. Many studies have shown that CAFs can promote tumor development by transmitting bidirectional signals to tumor cells via EVs (73). These CAF-derived EVs often contain death receptor ligands, such as PD-L1, and inhibitory cytokines, such as TGF-β (74). Specifically, EVs secreted by CAFs from breast tumors exhibit high levels of miR-92, which targets LATS2 and enhances the nuclear translocation of YAP2. This, in turn, promotes the interaction between YAP1 and PD-L1 enhancer regions, thereby promoting PD-L1 expression in breast cancer cells (75). Although the impact of CAF-derived EVs on immune cells in the TME remains unclear, considering that CAFs have been shown to regulate the tumor immune response and are associated with EVs, it is speculated that CAFs may play an essential role in tumor immunomodulation through EVs. In addition, further studies are planned to identify other possible targets for tumor prognosis assessment and drug research and development.

Mesenchymal stem cells (MSCs), as a kind of multipotent and self-renewable stromal cell, exist in many kinds of adult tissues. MSCs can migrate to tumors in large quantities; this phenomenon is known as MSC homing. Various factors, including chemokines, growth factors, and extracellular matrix components in the TME, enable this migration. MSCs are attracted to tumors by their interaction with cancer cells and the microenvironment, which influences tumor development. However, in addition to directly participating in tumor cell suppression or promotion through secreted factors, when MSCs become embedded within solid tumors, they undergo a series of phenotypic changes due to alterations in local cytokine stimuli. MSC-derived EVs (MSC-EVs) are secreted by MSCs and exhibit similar recruitment behavior and function as regulators of the tumor microenvironment. EVs transfer their contents such as specific proteins or genetic material, to adjacent cancer cells and the microenvironment. This transfer process can promote or inhibit tumorigenesis, angiogenesis, and metastasis by combining with specific microenvironmental cues and recipient cells (76). In addition, some studies have shown that some immunomodulatory properties of MSCs can be transferred through the EVs they secrete. A study has shown that hsa-miR-23b-3p in bone marrow MSC-EVs can maintain the balance of Th17 and Treg cells, thereby reducing the number of Th17 cells. This effect is achieved by targeting KLF5 to inhibit the PI3K/Akt/NF-κB signaling pathway (77). In contrast, EVs derived from adipose tissue MSCs also affect the function of CD11c+ DC cells by promoting the secretion of TGF-β and IL-10. This, in turn, increases the expression of DC co-stimulatory molecules and enhances the ability of DCs to regulate lymphocyte proliferation (78). In summary, MSCs are considered the best source of EVs, and EVs are believed to play a significant role in various functions of MSCs.

Neural stem cells (NSCs) are self-renewing pluripotent cells in the central nervous system that have the potential to differentiate into neurons, astrocytes, and oligodendrocytes (79). CNS diseases have caused many health issues, and NSCs have shown great potential as cell therapies (80). CNS diseases are serious health problems for which NSCs serve as an emerging cell therapy approach (80). Based on their tumor-homing properties, they can travel to primary tumors and regions where the tumor has spread, thereby serving as vehicles for drugs. As shown by Kortylewski et al., EVs obtained from NSCs carrying antisense oligonucleotides against STAT3 have shown more effective therapy for glioma than natural NSC EVs. By enhancing the immunoreactivity of human dendritic cells and mouse macrophages, it was determined that NSC EVs have good drug carrier potential in tumors (81).

## 3 EVs modulate anti-cancer drug resistance

**Figure 2:**
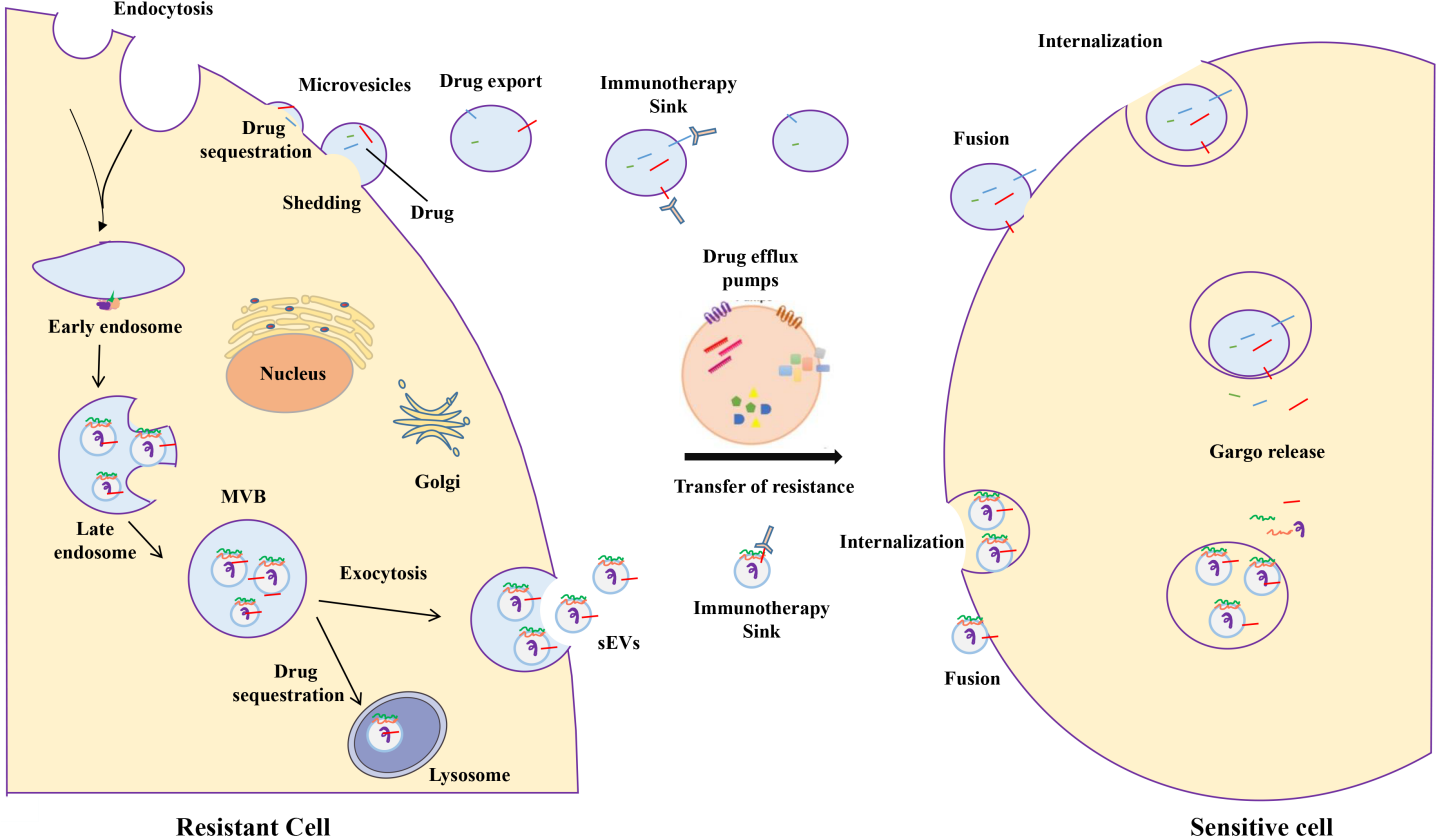
Mechanisms of extracellular vesicles-mediated transfer of anti-cancer drug resistance. Extracellular vesicles can mediate drug resistance by directly exporting or sequestering cytotoxic drugs, thereby reducing their effective concentrations at target sites. They may also compete with bona fide target cells for binding immunotherapeutic agents that target cellular antigens. Furthermore, extracellular vesicles facilitate the transfer of drug resistance to drug-sensitive cancer cells through the horizontal delivery of specific bioactive cargoes, including drug efflux pumps, pro-survival factors, inhibitors of apoptosis, and non-coding RNAs.

Drug resistance is a major concern in cancer treatment. It occurs when cancer cells do not respond to drugs intended to eliminate them. Many cancers initially respond well to these drugs; however, tumor cells slowly become resistant through various mechanisms that reduce their effectiveness. These mechanisms commonly include enhanced cell signaling to facilitate survival or prevent death, altered drug action on cells, overexpression of multidrug resistance (MDR) proteins, and increased activity of pumps to remove drugs from cancer cells. Recent reports have suggested that EVs play a role in drug resistance. EVs facilitate intercellular communication by transporting proteins and DNA/RNA to distant cells. Drug-resistant EVs are formed when the cargo confers resistance to the recipient cells. EVs confer resistance by directly exporting or trapping toxic drugs, thereby lowering the drug concentration at the target site. Moreover, EVs not only confer resistance but also transfer resistance traits to drug-sensitive cancer cells. They achieve this by horizontally transferring special bioactive molecules to recipient cells, thereby altering cell cycle regulation and death in recipient cells (**Figure 2**). Simultaneously, they facilitate communication between cancer and stromal cells in the tumor niche, promoting drug resistance and tumor progression. Understanding these resistance factors is crucial for developing new cancer treatments. The potential resistance mechanisms are detailed below.

### 3.1 EVs act as immunotherapy decoys

Cancer cells use EVs to reduce the effectiveness of anticancer agents. These EVs are coated with the same cellular antigens from which they originate and are arranged in the same manner. By showing the antigens targeted by immunotherapeutics on the cell surface, EVs act as baits for monoclonal antibody drugs, thereby decreasing the amount of drug available to attack tumors. For instance, in B-cell lymphoma, EVs present CD20 bait rituximab, an anti-CD20 monoclonal antibody, thereby lowering its availability to target cancerous B-cells (82). Similarly, laboratory and *in vivo* studies have revealed that HER2-positive EVs in breast cancer confer resistance to trastuzumab, an ant-HER2 antibody. EVs released from HER2-positive tumor cells or collected from the blood of patients with breast cancer bind to trastuzumab and block its effects (83). Recently, they have been implicated in drug resistance via immune checkpoint molecules. Immune checkpoint inhibitors, which won the 2018 Nobel Prize in Physiology or Medicine, have changed cancer immunotherapy by successfully stopping the messages sent by tumors to turn off T cell activity. Targeting the interactions between tumor cell checkpoint ligands, such as PD-L1, and inhibitory receptors on T cells, such as PD-1, can restore the host immune system to fight against cancer. However, not all patients can undergo this type of therapy. Early reports have indicated that many PD-L1-positive EVs linked to melanoma do not respond to anti-PD-1 treatments; therefore, such EVs can be used to determine treatment efficacy (84). EVs capture therapeutic antibodies on the outer layer of PD-L1 and remove drugs from tumor cells. PD-1 remains connected to T cells in tumors, leading to illness. This has also been observed in glioblastoma; laboratory studies have shown that TEVs with PD-L1 prevent T cell growth and antigen-fighting T cell production (85).

### 3.2 EV-mediated drug export and sequestration

Irrespective of whether anti-cancer drugs are administered systemically, orally, or via a shot under the skin, the goal is to deliver the drugs to the target tumor site. Drug entry into cells and cell wall penetration are important. However, abnormal tumor architecture, including improper blood vessel formation and altered events in and around cells, affects drug penetration and treatment efficacy (86). Cells that take up drugs can also force these drugs into the space around them with the help of special transporters that are part of the large MDR-linked ATP-binding cassette (ABC) transporter family (87). These efflux pumps decrease intracellular drug concentrations to levels that are too low to negatively affect cells. They also contribute to chemoresistance by using EVs to remove anticancer drugs from cancer cells. Sheddon et al. first showed a positive link between the expression of genes involved in vesicle release and drug resistance in different cell lines (88). Breast cancer cell lines have been used to provide first-hand evidence from direct experiments with fluorescence microscopy and flow cytometry, proving that doxorubicin is taken up by EVs and released outside the cells (88). Melanoma cells produce more EVs when acidified and actively transport cisplatin, making them cisplatin-resistant (89). Similarly, cisplatin-resistant ovarian cancer cells expel cisplatin via EVs (90). Furthermore, EVs derived from resistant cells carry the MDR-associated protein 2 (MRP-2) and the copper transport P-type ATPases ATP7A and ATP7B (90). B-cell lymphoma cells secrete EVs carrying doxorubicin and pixantrone in laboratory tests (91). ABCA3 is associated with EV formation and drug resistance. Blocking ABCA3, either genetically or pharmacologically, increases drug retention in cells (91).

Cancer cells neutralize chemotherapy by trapping drugs within intracellular vesicles, thereby preventing them from reaching the target site. In an MDR breast cancer model resistant to mitoxantrone, several EV-like structures containing the ABCG2 transporter and showing strong accumulation of mitoxantrone have been reported on the cell membrane (92). This type of drug sequestration in subcellular compartments has also been reported in leukemia cells resistant to multiple chemotherapeutic compounds. ABCA3 plays a major role (93): It is present on lysosomes and multivesicular body (MVB) membranes and traps cytostatics and prevents their escape into different compartments, thereby reducing their cytotoxicity (93).

### 3.3 EV-mediated transfer of drug efflux pumps

In addition to simply exporting or sequestering drugs, cancer cells can pass down drug resistance to daughter cells via the horizontal transport of EVs carrying drug efflux pumps. Drug efflux transporters belonging to the MDR ABC transporter family are important mediators of MDR in cancer cell lines (87). EVs harboring P-glycoprotein (P-gp, also called MDR-1/ABCB1), the most studied drug efflux pump, impart MDR to sensitive cells in various human cancers, such as prostate and ovarian cancers, acute T lymphoblastic leukemia, and osteosarcoma (94–97). For example, EVs isolated from the blood of patients undergoing docetaxel treatment were compared with those isolated from the same patients before treatment, and both were further applied to drug-sensitive and resistant prostate cancer cells. Notably, both drug-sensitive and drug-resistant cells showed responses that correlated with patient responses to the drug (94). Similarly, EV-mediated intercellular transfer of the functional MRP1 drug efflux transporter ABCC1 has been reported in leukemia (98). Other drug efflux transporters, such as ABCG2 and ABCA3, are also horizontally transferred through EVs, modulating drug resistance in recipient cells (99). Although many tumor cells produce EVs, whether the impact of the delivered cargo is maintained requires further investigation. The previous detection of selective P-gp/MDR-1 mRNA in EVs from doxorubicin-resistant osteosarcoma cells indicates that resistant tumor cells use several mechanisms to confer resistance, such as the transfer of MDR proteins or mRNAs containing instructions for producing MDR proteins. This makes drug resistance difficult to control, as it occurs via two different mechanisms (97). In contrast, the removal of drug efflux pumps via EV exocytosis makes tumor cells more sensitive to anti-cancer drugs *in vitro*. However, whether this seemingly beneficial effect observed *in vitro* leads to overall drug resistance within the TME *in vivo* requires further investigation. Additionally, EVs packed with MDR transporters can be absorbed by other cancer cells, such as tumor or stromal cells in the TME, possibly affecting their response to therapy.

### 3.4 EV-mediated export of pro-survival cargo

Cargo transfer by EVs contributes to the variability in tumor responses to anticancer treatments. This cargo includes pro-survival factors that improve cell survival and reduce sensitivity to apoptosis, thereby promoting resistance to anticancer drugs. EVs also contain elements associated with the phosphoinositide 3-kinase (PI3K)/AKT pathway, a key oncogenic signaling system involved in cancer cell development and survival. Hepatocyte growth factor, an important part of this pathway, was discovered in EVs derived from invasive hepatocellular carcinoma cell lines resistant to both *in vitro* and *in vivo* sorafenib treatment. This activates the hepatocyte growth factor/c-MET/PI3K/AKT signaling pathway (100). Platelet-derived growth factor receptor-β has been isolated from melanoma cells that have vesicles resistant to PLX4720. BRAF inhibitors are passed to recipient cells in a dose-dependent manner to ensure the activation of PI3K/AKT signaling and prevent BRAF inhibition (101). Triple-negative breast cancer is a recently discovered resistant subtype of breast cancer. Cell lines resistant to docetaxel and doxorubicin release EVs that impart chemotherapy resistance to non-tumorigenic breast cells (102). These EVs alter the expression of genes related to cell proliferation and death, including those associated with the PI3KG/AKT pathway, suggesting that these EVs bind to ligands or receptors. EVs can also contain pro-survival factors that affect immune functions, such as immune tolerance and escape. For example, TGF-β is a key agent in inducing immunosuppression. It is present in tumor-released EVs and inhibits IL-2 stimulation of peripheral blood lymphocyte proliferation in healthy donors via Treg induction (103). *In vivo* and *in vitro* studies on HER2-overexpressing breast cancer have revealed increased levels of the immunosuppressive cytokine TGF-β1 in EVs isolated from cells resistant to HER2-targeted therapy (104). Although the patient cohort was small and the results were inconclusive, plasma levels of EV-associated TGF-β1 possibly correlated with resistance to lapatinib and trastuzumab (104). Tumor cells become resistant to drugs and grow rapidly, avoiding cell death. The delivery of pro-survival factors via EVs is an additional method for tumor cells to avoid cell death induced by anticancer drugs. Survivin, a pro-survival protein belonging to the inhibitors of apoptosis family, has been detected in EVs from various tumor types (105–107). Survivin plays a role in preventing cell death and controlling mitosis. Therefore, recent studies have focused on survivin in cancer treatment (108). For example, Khan et al. demonstrated that EVs are involved in the stress-induced release of survivin from HeLa cells after sublethal proton irradiation (106). Recently, Kreger et al. reported that paclitaxel-treated aggressive MDA-MB-231 breast cancer cells release EVs that promote the survival of serum-starved and paclitaxel-treated fibroblasts and SKBR3 breast cancer cells (109).

The presence of miRNAs in EVs is linked to anti-cancer drug resistance in different cancer types (110–127) (**Table 3**). Studies on breast cancer and pancreatic ductal adenocarcinoma have shown that the EV-mediated transfer of miR-155 to drug-sensitive cells contributes to the development of chemoresistance. Interestingly, the increase in specific miRNA levels in EVs after chemotherapy may be a cellular mechanism to eliminate drug susceptibility-increasing miRNAs, thereby lowering their intracellular levels in cancer cells (124, 127).

**Table 3:** Extracellular vesicles miRNA cargo and chemoresistance in different cancers.

Cancer	Anti-cancer drugs	Cell lines	miRNA cargo	Mechanism	References
Lung	Cisplatin	A549/A549-DDP	↓ miR-100-5p	horizontal transfer	(110)
	Gemcitabine	A549/A549-GR	↑ miR-222-3p	horizontal transfer	(111)
	Cisplatin	A549/H1299	↑ miR-96	horizontal transfer	(112)
	Cisplatin	A549/A549-DDP	↓ miR-146a-5p	horizontal transfer	(113)
Breast	Docetaxel	MCF-7	↑ miR-100, miR-222, miR-30a, miR-17	horizontal transfer	(114)
	Tamoxifen	MCF-7	↑ miR-221/222	horizontal transfer	(115)
	Cisplatin 17-AAG PU-H71	Hs578Ts	↓ miR-134	horizontal transfer	(116)
	Doxorubicin Paclitaxel	MCF-7/MDA-MB-231	↑ miR-155	horizontal transfer	(117)
	Docetaxel Epirubicin Gemcitabine	MDA-MB-231/HMLE	↑ miR-1246	horizontal transfer	(118)
	Adriamycin	MCF-7/MCF-7-Adr	↑ miR-222	horizontal transfer	(119)
Oral cavity	Cisplatin	HSC-3/HSC-3R SCC-9/SCC-9R	↑ miR-21	horizontal transfer	(120)
Melanoma	Vemurafenib (PLX4032)	MML-1/MML-1R A375 PDX	↑ miR-211–5p	Autocrine	(121)
Glioblastoma	Temozolomide	SHG-44/U87MG	↑ miR-221	horizontal transfer	(122)
Prostate	Docetaxel	22Rv1/22Rv1RD DU145/DU145RD PC3/PC3RD	↓ miR-34a	horizontal transfer	(123)
Colon	Fluorouracil (5-FU)	DLD-1/DLD-1-5-FU	↑ miR-145, miR-34a	expulsion	(124)
Pancreas	Gemcitabine	Panc1/Panc1-GR	↑ miR-155	horizontal transfer	(125)
Leukemia	Imatinib	K562/K562-G01	↑ miR-365	horizontal transfer	(126)
	Daunorubicin	HL60/HL60AR	↑ miR-196, miR20a	expulsion	(127)

## 4 Conclusions and future perspectives

In conclusion, tumor and normal cells exhibit complex interactions, and EVs play a major role in these processes. These factors mainly promote immune evasion and suppression. TEVs specifically affect immune responses and reduce the effectiveness of immunotherapy (13). Normal cells, such as immune cells, release normal EVs that counteract these inhibitory effects, highlighting the importance of EV signaling in the immune response. During tumor interactions with the host, the outcomes depend on the balance between winning and losing in competition between the two. In addition to the application of EV–based tumor immunotherapy as an effective treatment method, the specific biological characteristics of EVs should be explored for tumor antigen detection and strong tumor immunity generation (128). EVs can also be used for drug delivery in cancer therapy. EV carrying tumor antigens, immune regulatory factors (inhibitory and activating), and immune cell chemokines are important for evaluating the patient’s immune state, examining treatment effects, predicting treatment prognosis, and evaluating overall prognosis (129). Drug resistance is a major obstacle in cancer treatment. Recently, EVs have gained significant attention as a novel mode of intercellular communication, particularly in liquid biopsy for cancer and for new therapeutic development, owing to their distinctive characteristics and vital cellular functions. A comprehensive understanding of the roles of EVs in drug resistance will facilitate the design of improved methods to regularly assess the treatment response of patients and restore the drug sensitivity of cancer cells.

Previous studies have reported positive preclinical findings, however, many problems must be resolved to facilitate the clinical application of EVs in monitoring therapies and treating resistant cancers. Despite their potential to predict treatment efficacy, no standardized scalable method has been established for the high–purity isolation of homogeneous EV populations. Current approaches for EV enrichment, isolation, and analysis are limited by their sensitivity, specificity, and reproducibility issues. High costs and poor separation efficiency remain key obstacles to the widespread commercial production of EVs for therapeutic use. Therefore, more convenient, sustainable, and cost–effective analysis platforms are urgently needed to facilitate the use of EVs as reliable biomarkers of drug resistance. Notably, EVs possess different biological properties depending on their origin, which alters the specific cancer that can be recognized by EV–based markers. The need to quickly and efficiently isolate EVs from complex biological liquids, such as blood and other body fluids, to facilitate their detection with high sensitivity, specificity, and accuracy remains a major hurdle for their clinical application. Natural and intrinsic biological substances exhibit the same characteristics as EVs as drug carriers. EVs show potential for overcoming cancer drug resistance by delivering small interfering RNAs, miRNAs, chemotherapeutics, and other agents (130). However, their application in hospitals is limited. Many limitations need to be addressed to facilitate their use in overcoming drug resistance in clinics. These include determining the optimal drug administration route, drug delivery site, and action duration, assessing safety, identifying the optimal drug dose, exploring drug loading and engineering, establishing large–scale development methods, and assessing quality. Although substantial experimental evidence supporting the role of EVs in drug resistance has been obtained using preclinical models, most studies have evaluated only limited patient cohorts. Moreover, although EV–based therapies have been assessed in patients with cancer since the 2000s, standards and protocols for the production of clinically compliant EV–based therapeutics and their quality control have not yet been established. Therefore, designing and conducting clinical trials that adhere to all relevant regulations is crucial for facilitating cancer treatment with EVs.

In conclusion, research on EVs is ongoing and continuously increasing our knowledge of tumor immunobiology, showing great promise for the development of new treatment methods, especially in precision and personalized medicine. Future investigations should address the existing scientific, technical, and translational challenges to fully realize the potential of EVs in oncology.
